# Supplementary material for: RNA structure prediction using positive and negative evolutionary information
Source: PLoS Comput Biol. 2020 Oct 30;16(10):e1008387. doi: 10.1371/journal.pcbi.1008387 (PMC7657543; doi:10.1371/journal.pcbi.1008387)
Supplement: S1 File — Includes alignments, and source code. (GZ) [file pcbi.1008387.s008.tar.gz › supplemental_material/src/rscape_v1.5.4.tar.gz!/rscape_v1.5.4/lib/infernal/infernal-1.1.2/documentation/userguide/rigfilters_doc.html]

README for cm2hmm


# README for cm2hmm

This program implements the techniques described in Z. Weinberg and W.L.
Ruzzo (2004) "Faster genome annotation of non-coding RNA families without loss
of accuracy", in *Proc. Eighth Annual International Conference on Research in
Computational Molecular Biology (RECOMB),* ACM Press, 243-251.

## Licensing

The source code for cm2hmm and cm2hmmsearch is
copyright 2004 by Zasha Weinberg and distributed under the BSD license.

The source depends on the cfsqp 3rd-party library.  CFSQP is
distributed by www.aemdesign.com. 
It is not freely available, but is available on request for free for
academic institutions.

## Installation

Installation is handled as an integrated part of the Infernal package.

To include rigorous filters when building Infernal, first configure the package

```
./configure --with-rigfilters --with-cfsqp=/path/to/cfsqp
```

The first option specifies that cm2hmm should be compiled (off by default), and the
second specifies the location of the cfsqp source code. Alternatively, you may
copy the source code into infernal-x.xx/rigfilters/cfsqp/ and omit the second
option.

Following a successful configure, type

```
make
```

## Usage

cm2hmm creates a compact- or expanded-type HMM from a given CM. 
cm2hmmsearch searches a FASTA sequence file using a CM and profile HMM
rigorous filters created using cm2hmm.  Both programs display simple usage
instructions when run without any parameters.

The input format for cm2hmm is:

```
cm2hmm <input CM file name> <output HMM file name> <0th-order Markov model specification> <HMM type & output format> <solver specification>
        <input CM file name> : file name of a CM in Infernal format.
	<output HMM file name> : file name of HMM to create.
        <0th-order Markov model specification> : one of the following:
                uniform : use a uniform 0th-order model (all nucleotides have probability 0.25)
                gc <fraction> : the G+C content is <fraction>, a number from 0 to 1.
                file <file name> : load it from a file (logic to create these files from an input sequence may or may not be implemented in distribution.
        <HMM type & output format> : one of the following:
                compact : create a compact-type profile HMM in the default text format.
                expanded : create an expanded-type profile HMM in the default text format.
        <solver-specification> : one option currently:
		cfsqp <B> <C> : use CFSQP, sending solver parameters B&C.  <B>=0, <C>=1 are reasonable parameters.  Refer to the CFSQP manual for details.
		
```

The input format for cm2hmmsearch is:

```
cm2hmmsearch <window len> <score threshold> <CM file name> <compact profile HMM file name> <expanded profile HMM file name> <sequence file> <run CM?>
        <window len> : window length parameter for CM scan.
        <score threshold> : hits below this threshold will be ignored (and likely filtered out by the profile HMMs).
        <CM file name> : file name of a CM in Infernal format.
        <compact profile HMM file name> : name of a profile HMM to do filtering, or "-" (a single dash) to not use this HMM.  Although this HMM is presumed to be compact type, this is not enforced.
	<expanded profile HMM file name> : similar idea to previous field, but for the expanded profile HMM.
        <sequence file> : name of a sequence file in FASTA format.
        <run CM?> : if "0" do NOT actually run the CM, just do the filtering and report the filtering fraction.  If "1", run the CM to find hits.
		
```

Here's an example of creating both compact- and expanded-type HMMs for RF00095,
and scanning the *Pyrococcus abyssi* genome.

From infernal-x.xx/rigfilters/cm2hmm, enter the following commands (which each take a minute or so to complete):

cm2hmm data/RF00095.cm data/RF00095\_compact.hmm file data/Ecoli\_0mm.mm
compact cfsqp 0 1

cm2hmm data/RF00095.cm data/RF00095\_expanded.hmm file
data/Ecoli\_0mm.mm expanded cfsqp 0 1

cm2hmmsearch 150 23.5 data/RF00095.cm data/RF00095\_compact.hmm
data/RF00095\_expanded.hmm data/AL096836.fna 1

The first two commands create the HMMs given the CM in data/RF00095.cm. 
They are both optimized based on a 0th-order Markov model of the *E. coli*
K-12 genome.  The last command uses these HMMs to accelerate a search of
the *Pyrococcus abyssi* genome (data/AL096836.fna).  The
search outputs the family members found in basically the same format as
Infernal.  An important new piece of information is the 'frac let thru so
far', which gives the filtering fraction measured on this genome.  The
reported filtering fraction is for the 2nd HMM, i.e. the expanded-type
one.  (2d-fracLetsThru is a measure of the filtering fraction that
attempts to reflect the fact that the dynamic programming algorithm for CMs has
an extra dimension, so the filtering fraction is a somewhat pessimistic
estimate of the actual speed-up).

### What 0th-order Markov model to use?

The choice of Markov model in the infinite-length forward algorithm does not
usually affect the filtering fraction that much, but a good choice
can yield a modest improvement in filtering fraction (typically
around 10%).  In general, it's best to use the 0th-order model of the
genome that has the highest (worst) filtering fraction.  To estimate this,
create a compact-type HMM from any model, and run it on the *Bordetella*,
*E. coli* and *S. aureus* genomes.

### Using compact- or expanded-type HMMs, or both

Once you've picked a 0th-order Markov model, the easiest thing to do is to
create both compact- and expanded-type HMMs, and run them on the three
genomes.  This yields an estimate of the filtering fraction for the two
HMMs.  If the filtering fraction of the compact-type HMMs is above 0.25,
it's probably not worth using it (this is based on a rule of thumb that the
expanded-type HMM runs 30% slower than the compact-type HMM, so if the
compact-type fraction is above 0.25, it's not worth using it).  If the
compact-type HMM filtering fraction is low, there's no need to use the
expanded-type HMM, but it can't hurt.

The difference in speed between the CM and the HMMs is mainly dependent on the
window length W.  The HMM is faster than the CM by a factor of usually a
bit over W.  So, if the filtering fraction is significantly below 1/W,
then the search time is dominated by the HMM's search time, and there's no
point in getting a better filtering fraction.
